# Supplementary figures and images for: Identification of enzymes that have helminth-specific active sites and are required for Rhodoquinone-dependent metabolism as targets for new anthelmintics
Source: PLoS Negl Trop Dis. 2021 Nov 29;15(11):e0009991. doi: 10.1371/journal.pntd.0009991 (PMC8659336; doi:10.1371/journal.pntd.0009991)

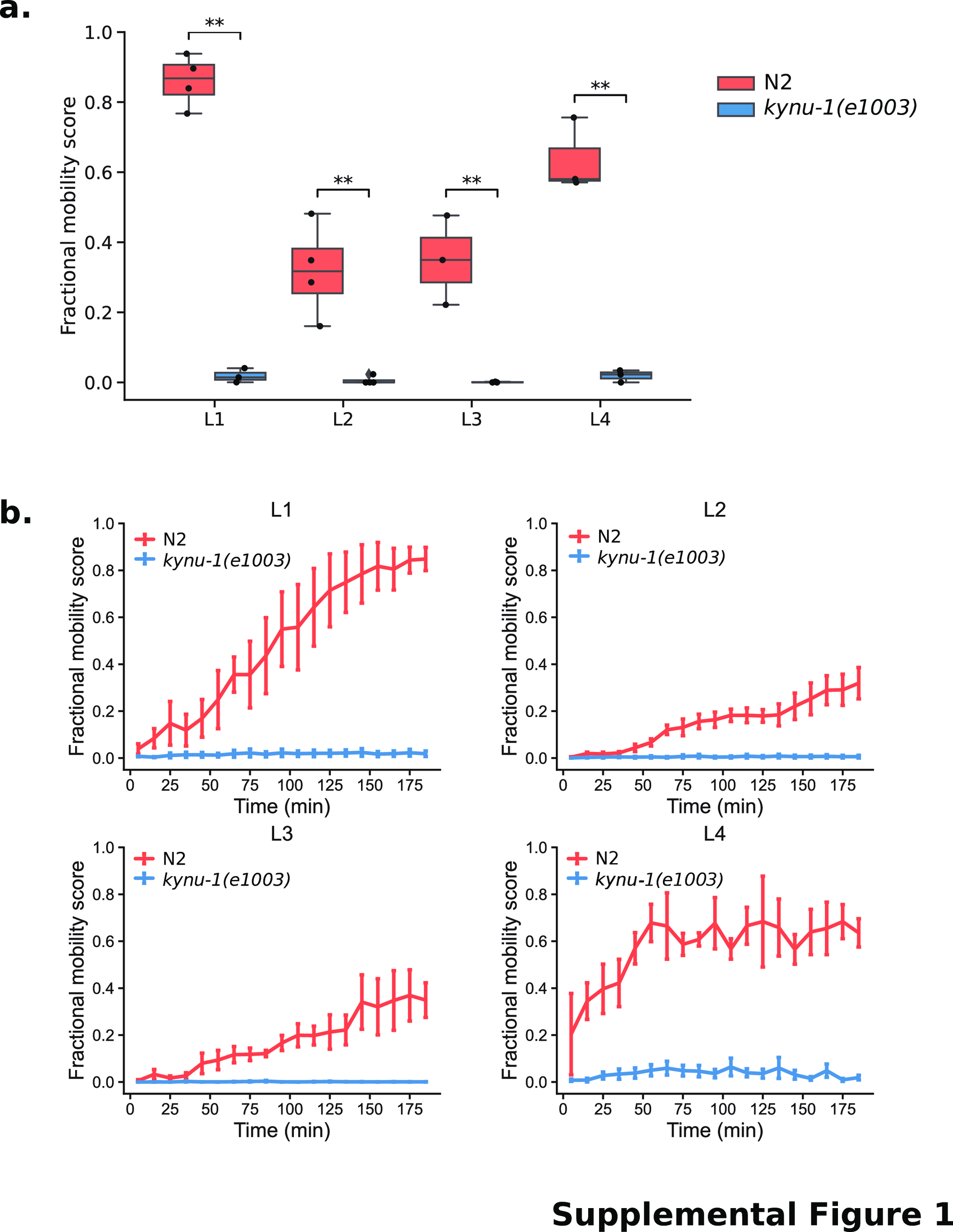

Supplement: S1 Fig — (a) RQ is required for survival following extended treatment with KCN at all C. elegans larval stages of development. Wild-type (N2, red boxes) and kynu-1(e1003) mutant animals (blue boxes) at various stages were exposed to 200 μM KCN for 15 h. KCN was then diluted and worm movement was measured after a 3 h recovery. Boxplots show the worm mobility scores 3 h after recovery from KCN and comprise 3–4 independent biological replicates. Significance of difference between N2 and kynu-1(e1003) was calculated using a t-test (** = p < 0.01). (b) Movement of N2 (red curve) and kynu-1(e1003) (blue curve) worms for the 3 h period after recovery from 200 μM KCN. Curves show the mean of 3–4 biological replicates and error bars represent standard error. (TIF) [file pntd.0009991.s001.tif]

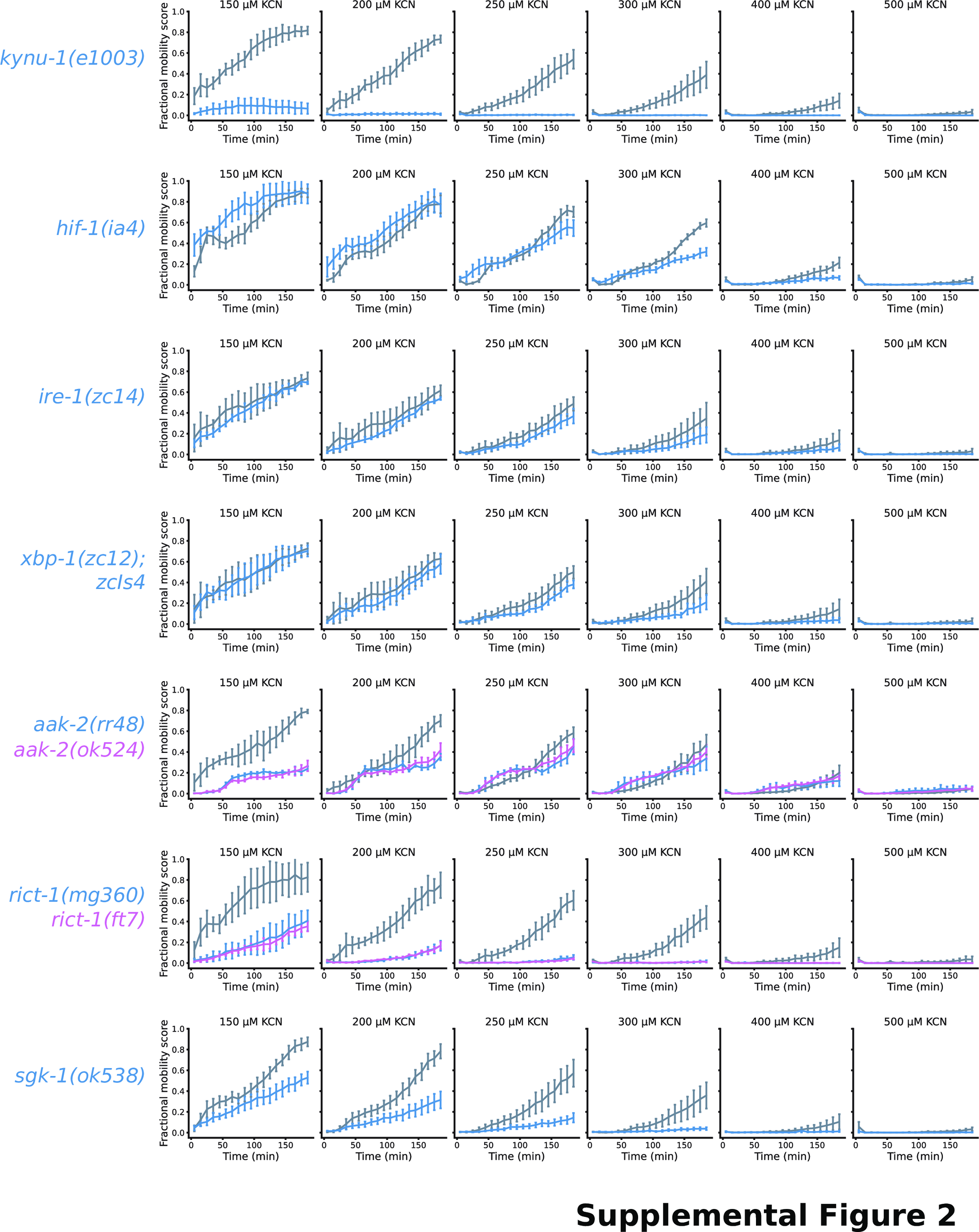

Supplement: S2 Fig — Wild-type (N2, grey curve) and mutant worms (blue and purple curves) defective in components of the hypoxia pathway were exposed to 150–500 μM KCN for 15 h. KCN was then diluted 6-fold and worm movement was measured over 3 h to track recovery from KCN exposure. (TIF) [file pntd.0009991.s002.tif]

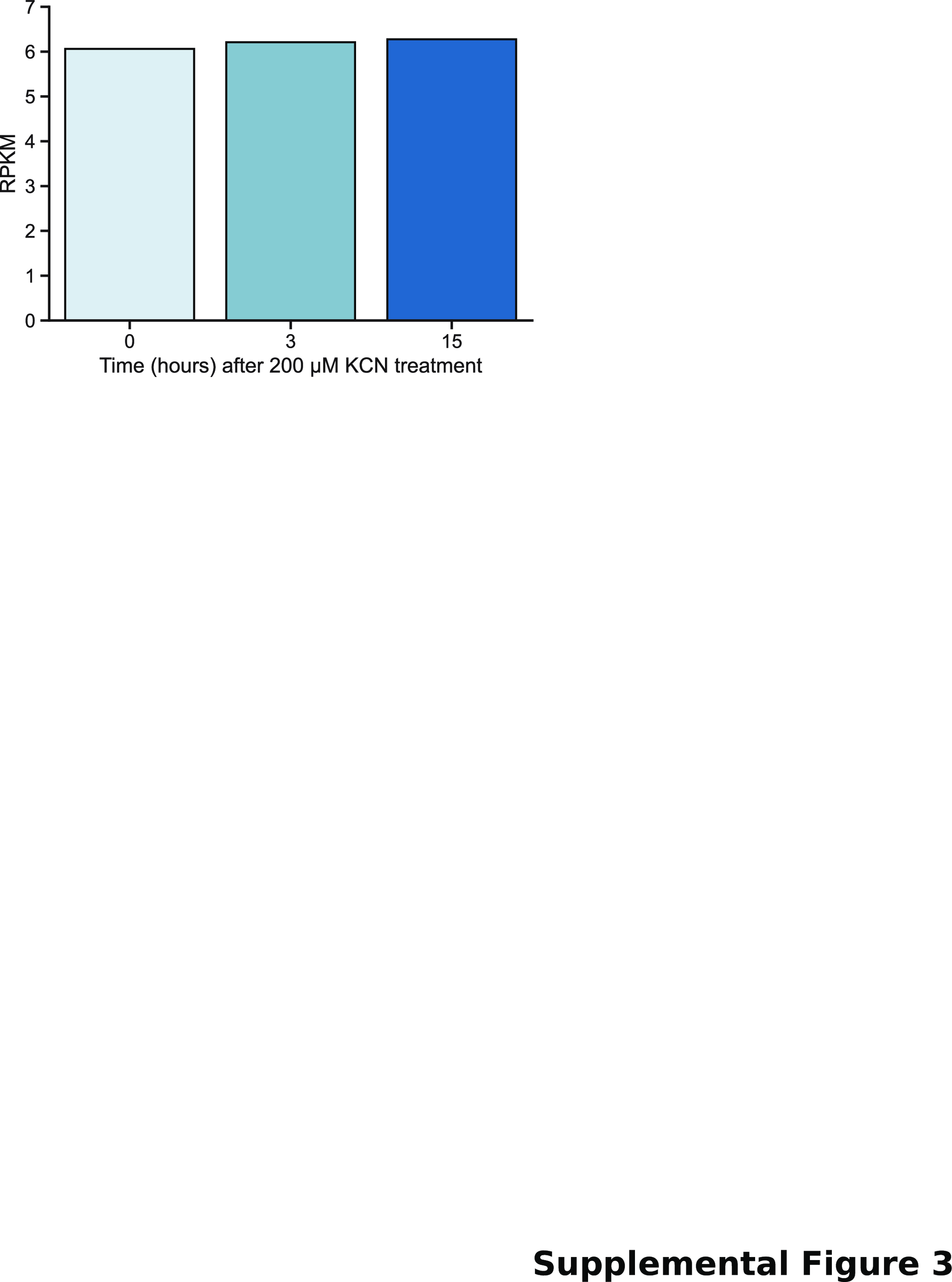

Supplement: S3 Fig — Expression levels (RPKM) of nhr-57 from RNA-seq data. L1 C. elegans worms were treated with 200 μM KCN and samples were collected 0, 3 and 15 hours after incubation with KCN. (TIF) [file pntd.0009991.s003.tif]

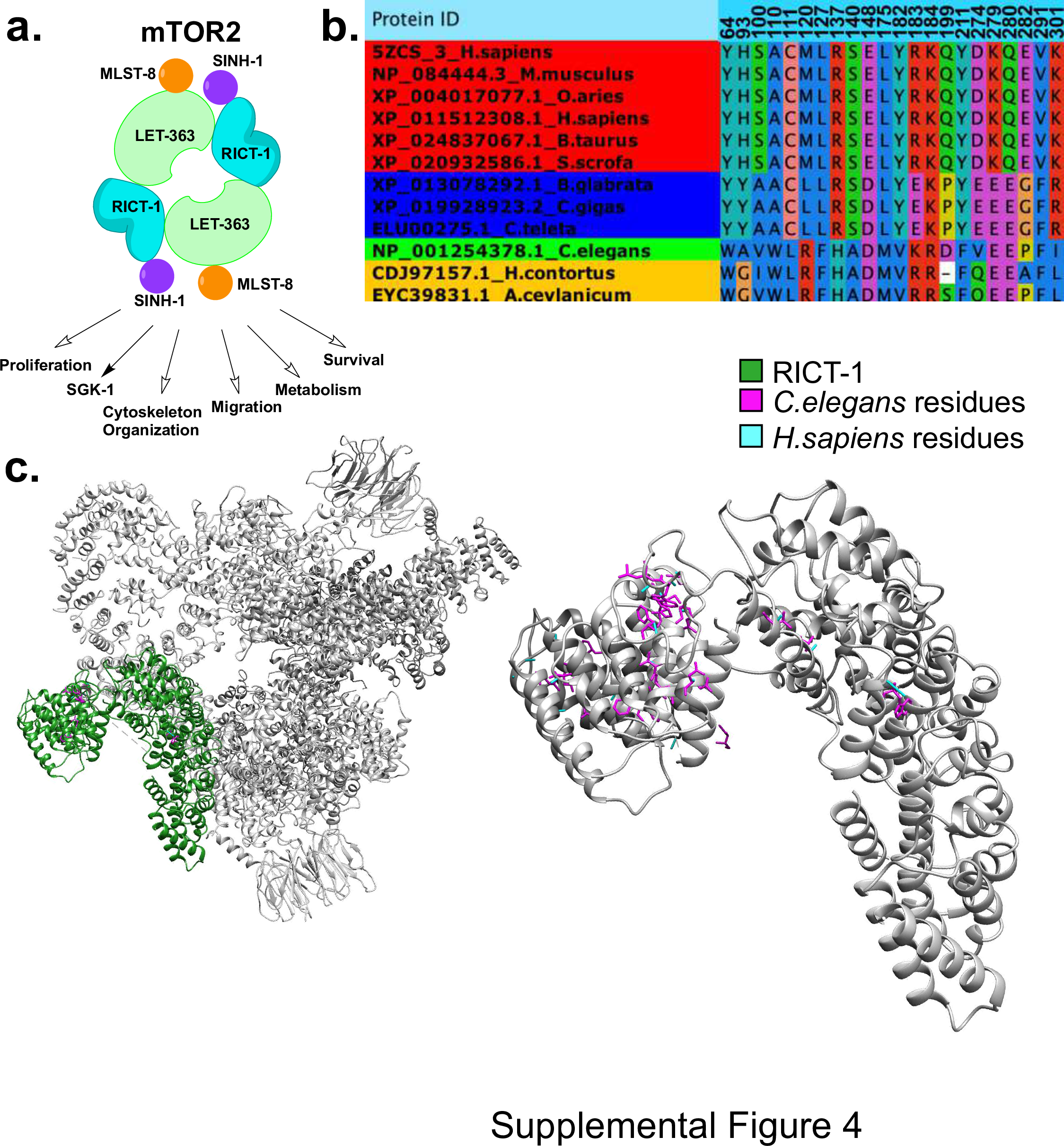

Supplement: S4 Fig — (a) RICT-1 is a component of the mTOR2 complex which regulates a number of downstream processes including SGK-1. (b) Conserved divergent residues of RICT-1 with a cutoff of 2 using S3Det [38] shows conserved divergence though much of it is not RQ-specific. (c) Crystal structure of human mTORC2 (PDB: 5ZCS) as visualized in Chimera [85] with RICT-1 in green. C. elegans sequence (pink residues) are threaded over by homology but are dispersed throughout the protein and seem more likely to affect its dynamics than be druggable. (TIF) [file pntd.0009991.s004.tif]

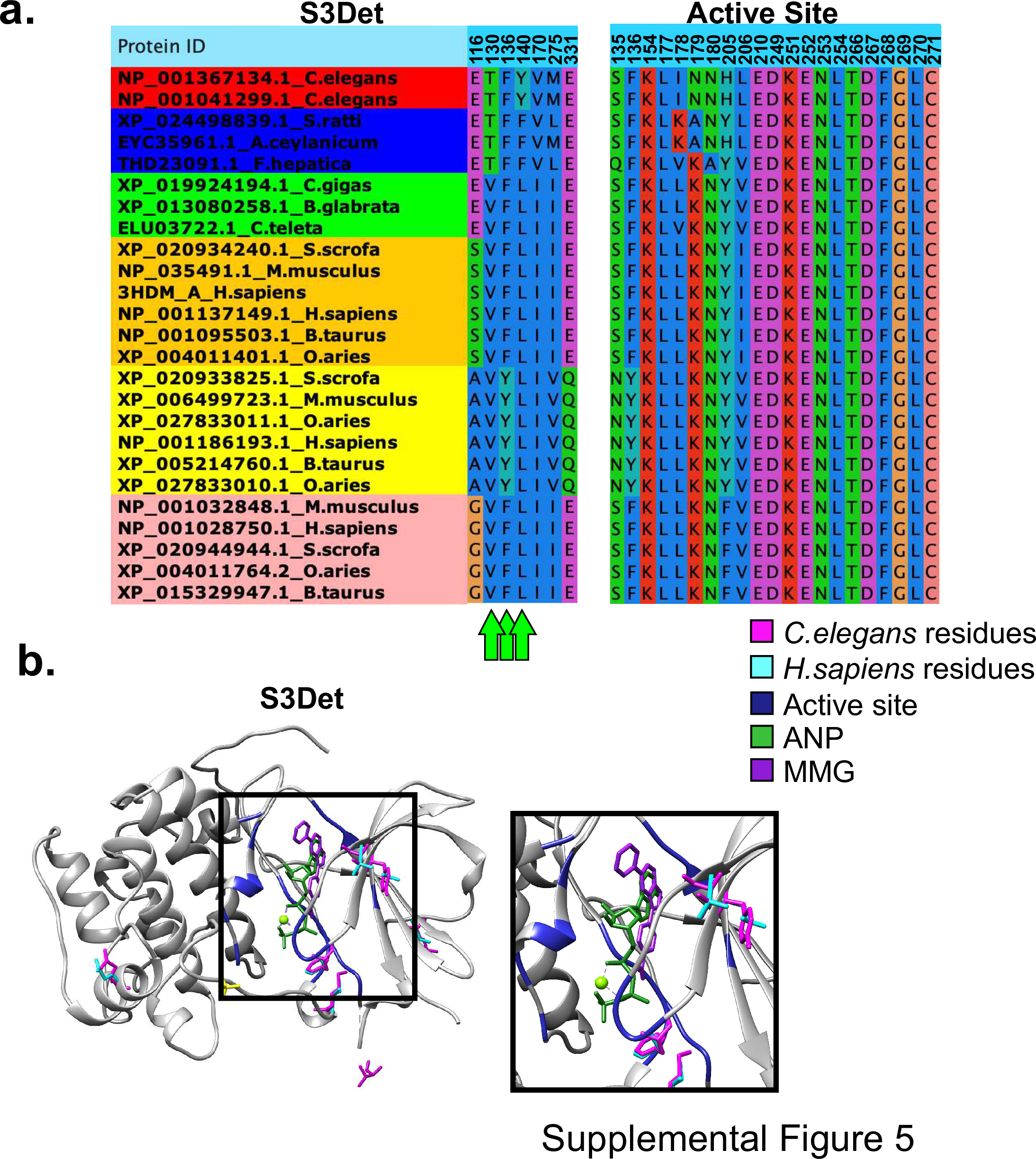

Supplement: S5 Fig — (a) Conserved divergent residues of SGK-1 with a cutoff of 2 using S3Det [38] shows conserved divergence though much of it is not RQ-specific but rather reflects the SGK1/2/3 variation within hosts. The same is true of the previous characterized active site [76,77]. (b) Crystal structure of human SGK-1 (PDB: 3HDM/2R5T) as visualized in Chimera [85]. C. elegans sequence (pink residues) are threaded over by homology. (TIF) [file pntd.0009991.s005.tif]

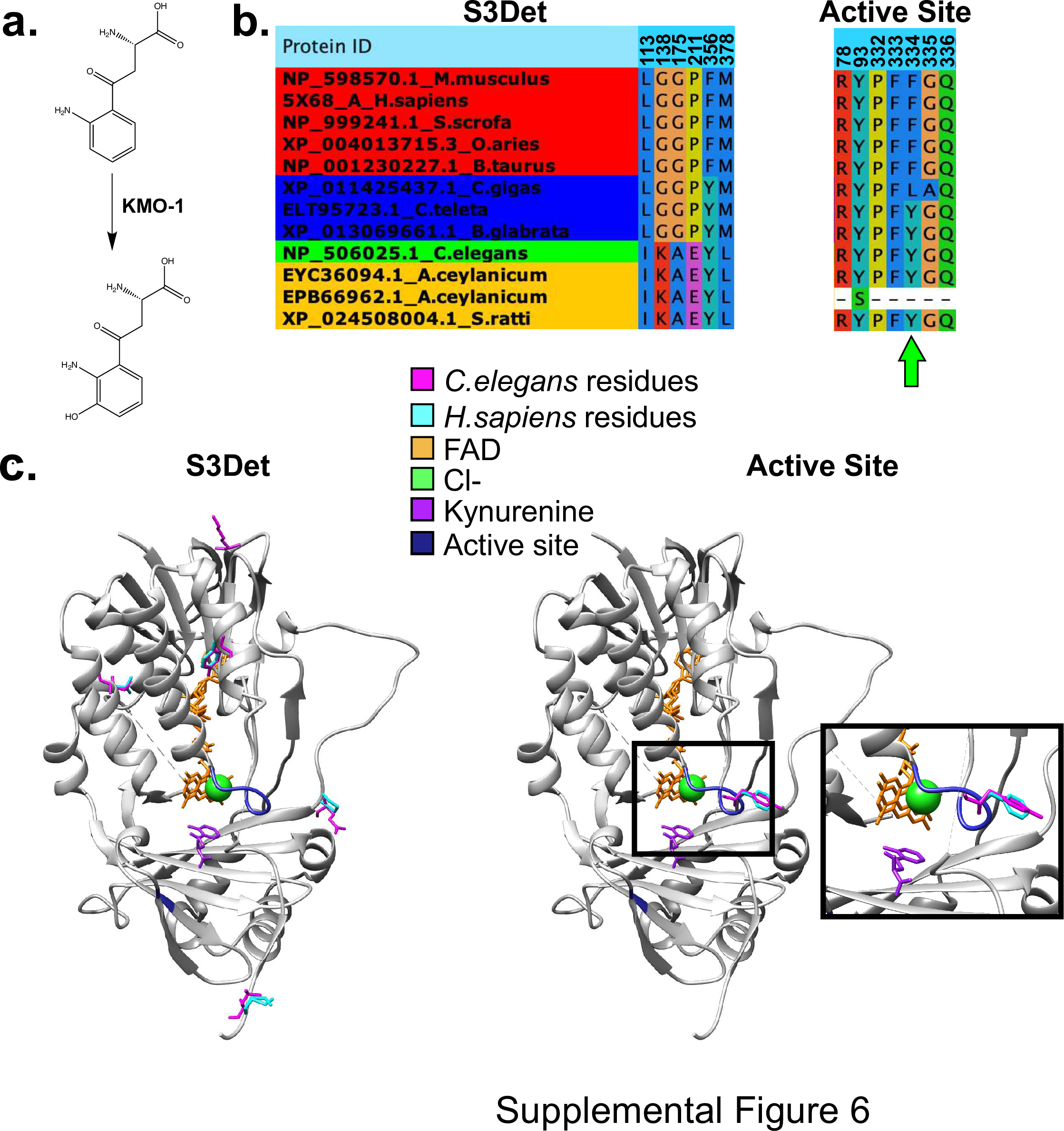

Supplement: S6 Fig — (a) KMO-1 catalyzes a key reaction in the biosynthetic pathway which makes the 3HA, a RQ precursor. (b) Conserved divergent residues of KMO-1 with a cutoff of 2 using S3Det [38] shows poor conservation amongst helminths while the known active site [157] does have a conserved divergent residue (green arrow). (c) Crystal structure of P.fluorescens KMO-1 (PDB: 5NAK) and H.sapiens KMO-1 (PDB: 5X68) as visualized in Chimera [85] with C. elegans sequence (pink residues) threaded over by homology. A single conserved divergent residue is within the substrate binding active site (blue). (TIF) [file pntd.0009991.s006.tif]

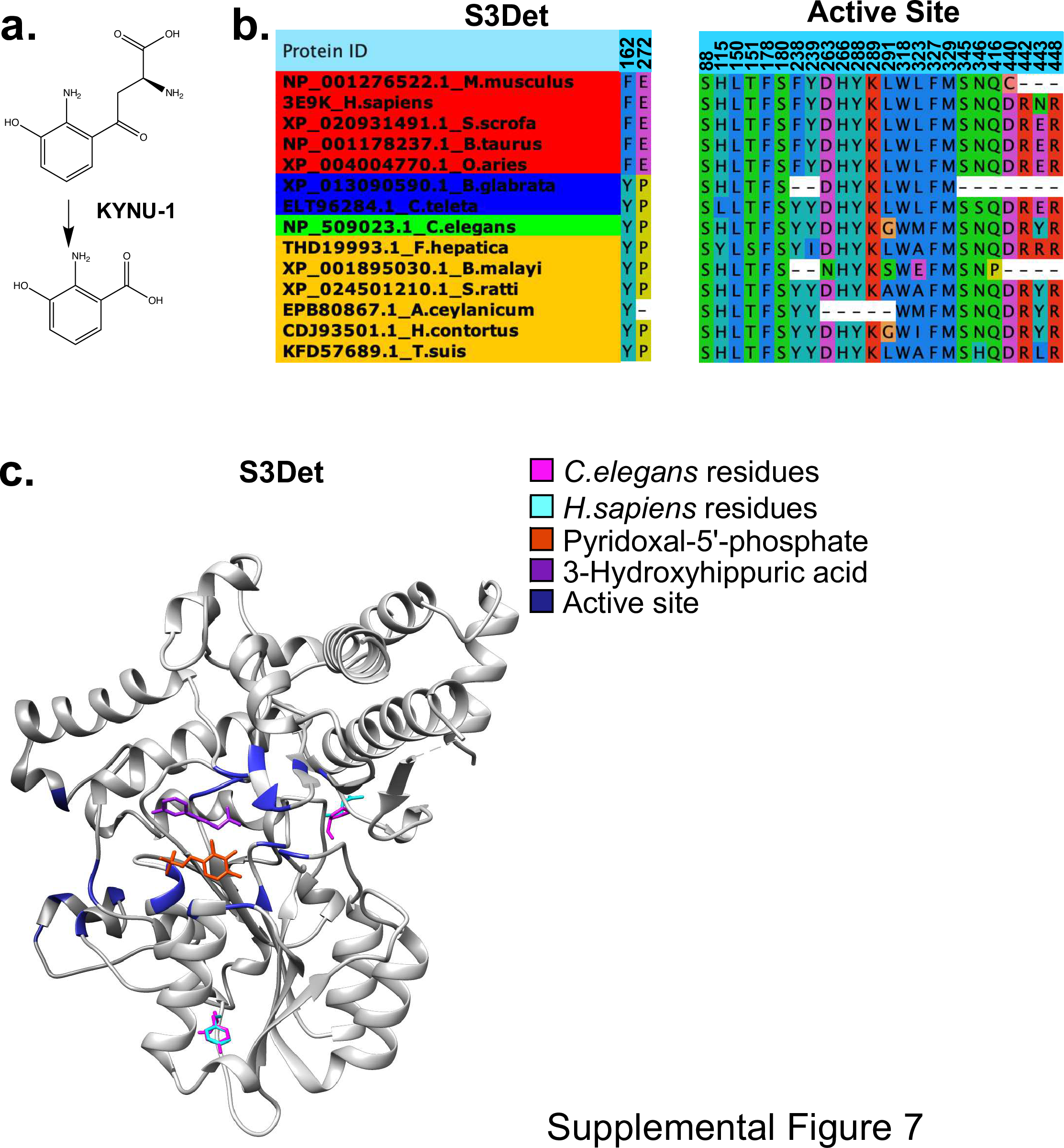

Supplement: S7 Fig — (a) KYNU-1 catalyzes a key reaction in the biosynthetic pathway which makes the 3HA, a RQ precursor. (b) Conserved divergent residues of KYNU-1 with a cutoff of 2 using S3Det [38] shows strong conserved divergence while the known active site [158] does not. (c) Crystal structure of human KYNU-1 (PDB: 3E9K) as visualized in Chimera [85] with C. elegans sequence (pink residues) threaded over by homology. (TIF) [file pntd.0009991.s007.tif]

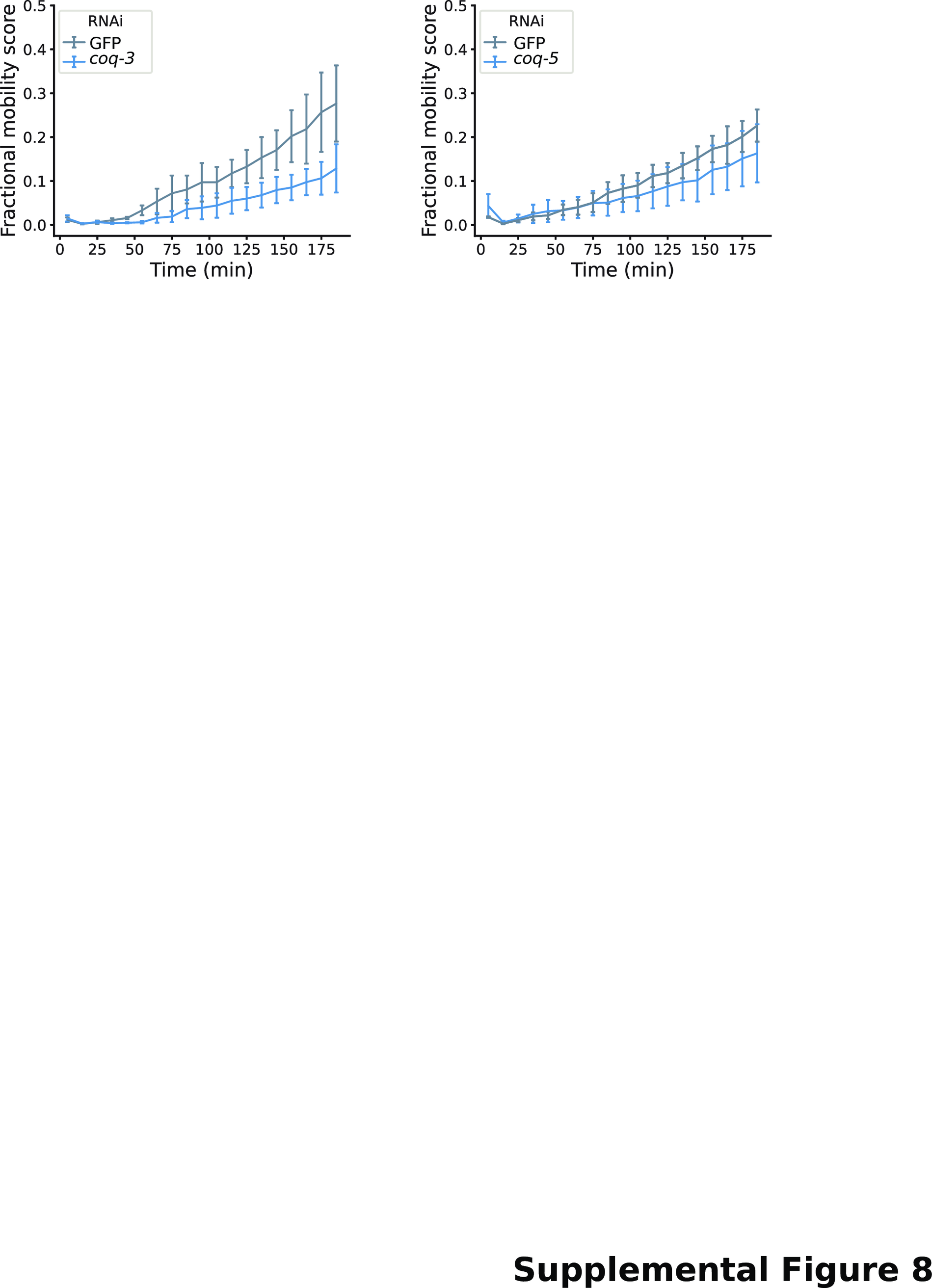

Supplement: S8 Fig — RNAi knockdown of coq-3 and coq-5 was performed in L1 worms and after one generation their L1 progeny were subjected to 15 h 200 μM KCN treatment followed by a 3 h recovery (blue curve, see Materials and methods). An RNAi clone targeting GFP (grey curve) was used as a negative control. Curves show the mean of 3 biological replicates and errors bars represent standard error. (TIF) [file pntd.0009991.s008.tif]

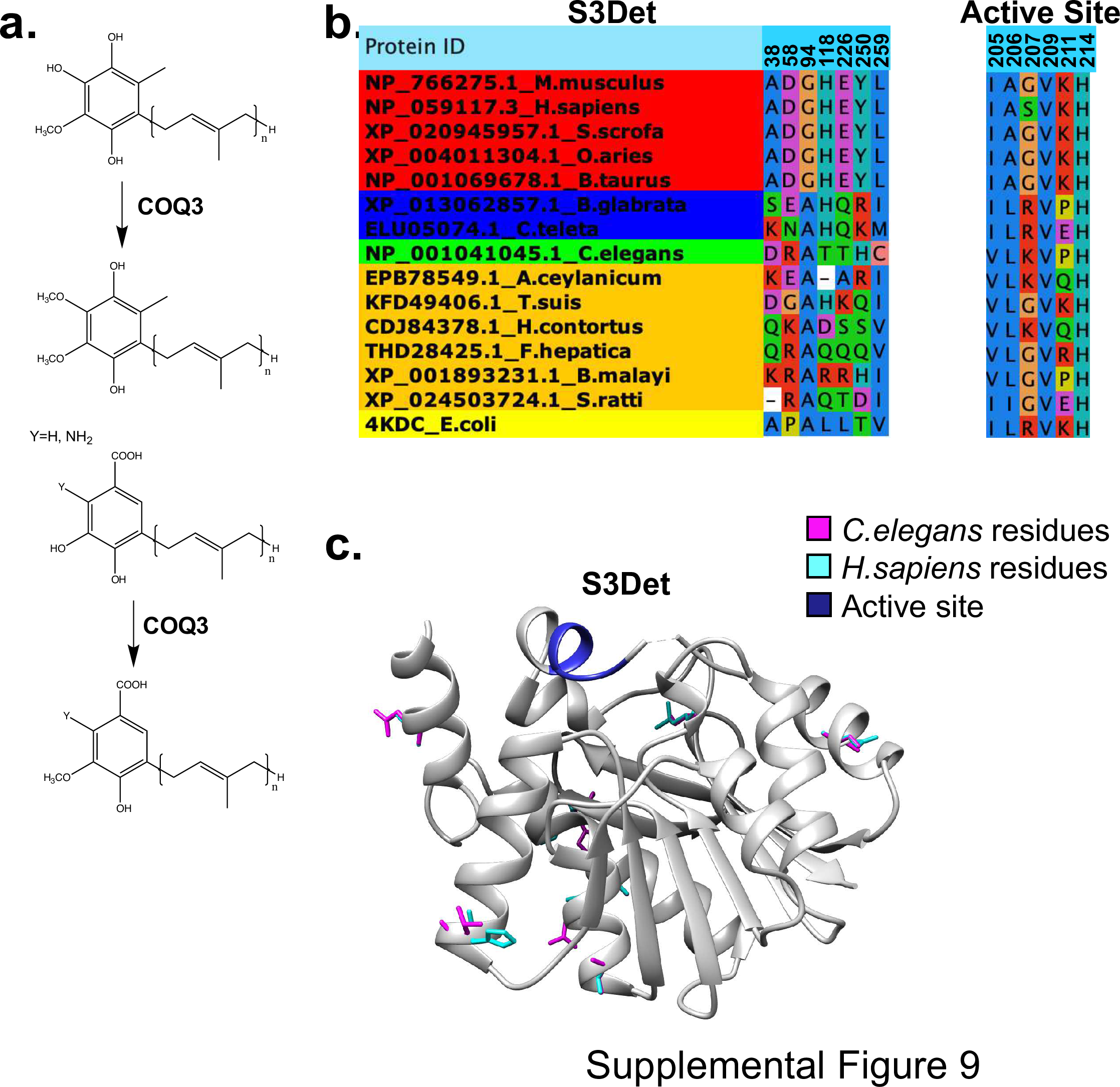

Supplement: S9 Fig — (a) COQ-3 catalyzes several reactions in the UQ/RQ biosynthetic pathway. (b) Conserved divergent residues of COQ-3 with a cutoff of 2 using S3Det [38] shows poor conservation amongst helminths while the known active site [159] is either conserved or very flexible in helminths as with 211. (c) Crystal structure of E.coli COQ-3 (PDB: 4KDC) as visualized in Chimera [85] with C. elegans sequence (pink residues) and H.sapiens sequence (cyan residues) threaded over by homology. Conserved divergent residues are not near the active site (blue). (TIF) [file pntd.0009991.s009.tif]

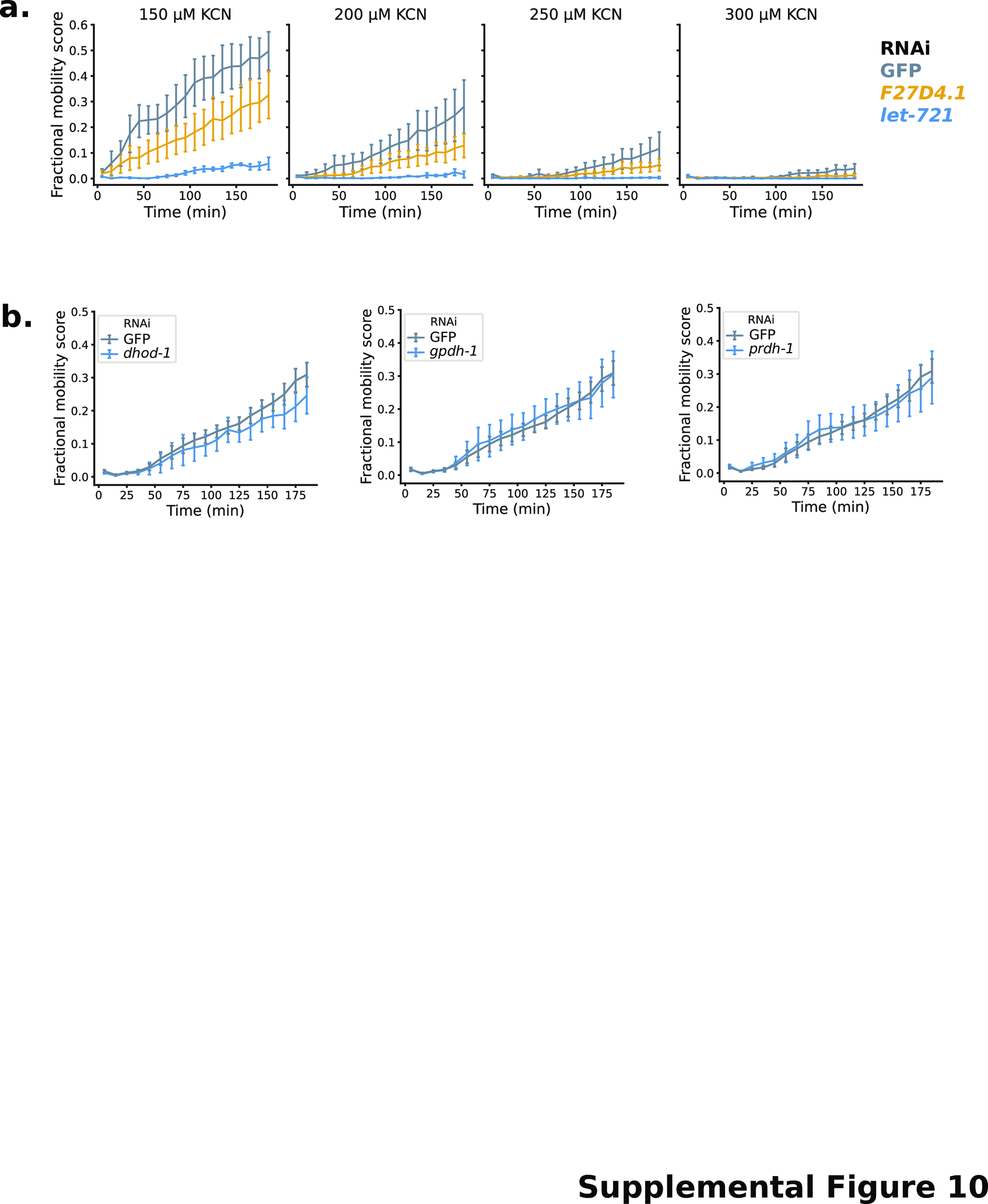

Supplement: S10 Fig — (a) RNAi knockdown of the ETF subunit alpha gene F27D4.1 (yellow curve) and the ETFDH gene let-721 (blue curve) was followed by a 15 h KCN treatment at various concentrations and recovery of movement was tracked for 3 h (see Materials and methods). An RNAi clone targeting GFP (grey curve) was used as a negative control. (b) RNAi knockdown of various other QDH genes (blue curve) was performed as described above and worms were subjected to 15 h 200 μM KCN treatment. An RNAi clone targeting GFP (grey curve) was used as a negative control. All curves show the mean of at least 3 biological replicates and error bars represent standard error. (TIF) [file pntd.0009991.s010.tif]

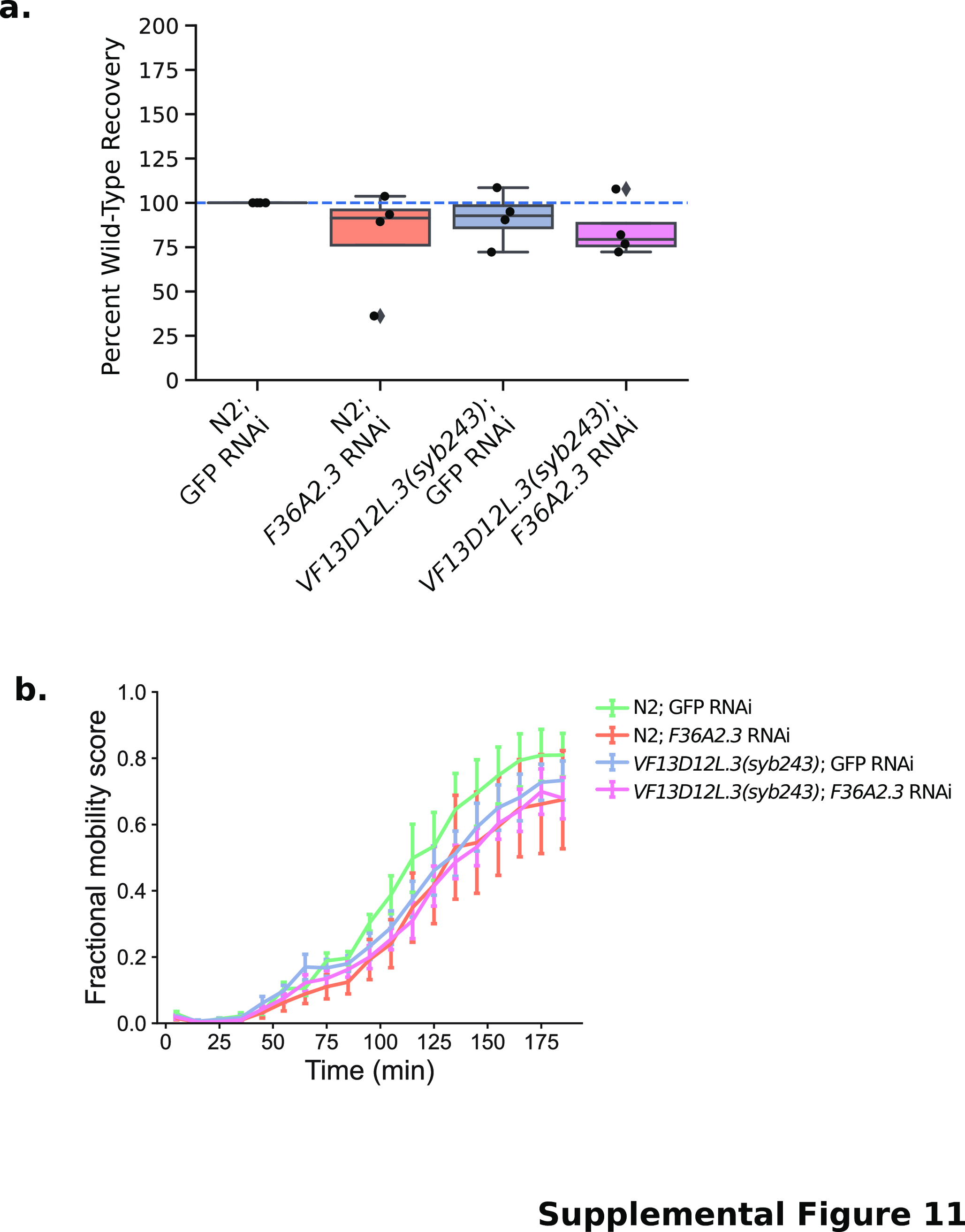

Supplement: S11 Fig — (a) Effect of loss of archaeal MDH genes on the ability of C. elegans worms to survive extended KCN exposure. RNAi was performed in either wild-type or VF13D12L.3.1(syb243) L1 worms and their progeny were subjected to 15 h of 200 μM KCN treatment followed by a 3 h recovery from KCN treatment (see Materials and methods). RNAi targeting GFP was used as the negative control. Boxplots show the mobility scores of 4 independent biological replicates after 3 h recovery period and are normalised relative to the GFP control in wild-type N2 worms. The RNAi and mutant combination shows no significant difference in recovery using a two-way ANOVA test (p = 0.52). (b) Worm movement during the 3 h recovery period from KCN. Curves show the mean of 4 biological replicates and error bars represent standard error. (TIF) [file pntd.0009991.s011.tif]
