# Supplementary material for: Identification of enzymes that have helminth-specific active sites and are required for Rhodoquinone-dependent metabolism as targets for new anthelmintics
Source: PLoS Negl Trop Dis. 2021 Nov 29;15(11):e0009991. doi: 10.1371/journal.pntd.0009991 (PMC8659336; doi:10.1371/journal.pntd.0009991)
Supplement: Attachment — Submitted filename: response_pointbypoint_ML_JT2.docx [file pntd.0009991.s036.docx]

We thank all three reviewers for their comments and have addressed their comments point by point below. In particular we realise that we were lax in our use of nematode, STH, and were imprecise in how referred to hosts and have tried hard to correct this throughout.

The principal changes that we have made are:

(1) all the data presented now have at least 3 independent biological replicates and all statistical analyses are shown. Note that in every biological replicate there are multiple technical replicates and that each technical replicate measures the phenotype in 100-150 animals so in total each measurement is derived from ~1000 animals.
(2) All the trees have been removed.
(3) We were very lax in using *C. elegans,* nematode, and STH almost interchangeably and thank all reviewers for pointing this out. We have tried hard to tighten this up and have added sentences indicating that all functional data derive from *C. elegans,* a free-living nematode. The active site analysis we focused around comparing STHs with their mammalian hosts and hope we have stated that clearly throughout.
(4) We added a time course analysis showing the effects of KCN and the ability of worms to recover at different stages of the life cycle.
(5) We re-organized Fig 2 especially to keep pathways separate and understand that was a little confusing.
(6) At times there were differences in views between reviewers and we tried to tread a middle line.
(7) We have added text to indicate that the goal of this study was to identify targets for possible anthelmintic drugs and not to suggest that every single drug predicted *in silico* against these targets would have ideal pharmocokinetics or would have no off-target effects and hope this is now clearer.

We hope the reviewers feel that the paper is improved and that they now feel it is suitable for publication.

**Point-by-point response to reviewers**

**Methods**

**Reviewer #1: No. As described below, the manuscript is difficult to read for flow and results. Some data require more replicates (only two or three data points are presented with no description of how independent they are). Statistical differences can not be significant with so few replicates in some comparisons.**

*All data now have three independent biological replicates at the minimum. We note that each biological replicate is the average of multiple technical replicates, each of which reflect the movement of ~150 worms. Statistics have been updated for all the experiments in the paper and we have updated the text to reflect this. We are now more precise in the comparisons that we make and hope that this satisfies the reviewer. For example, we now note in the text:*

*“aak-2 has a 50-60% reduction in recovery (1.00e-04 < p <= 1.00e-03) and rict-1 has a 75% reduction (1.00e-04 < p <= 1.00e-03).”*

*“We found that sgk-1 has a variable (1.00e-02 < p <= 5.00e-02) but roughly 50% reduction in recovery which suggests it is also partially required for C. elegans to use RQ-dependent metabolism”*

**Reviewer #2: The phylogenetic trees are inaccurate -  there is no way that highly conserved proteins like AMPK that where present in the common ancestor of all the phyla you examine do not have a molecular phylogeny that mirrors the phylogenetic tree of the phyla those proteins were taken from (e.g. echinoderms and mammals are deuterostomes and should share a clade, likewise arthropods and nematodes). Fortunately, the trees don't really contribute anything to your conclusions and are not necessary for the important data, which are the sequence alignments, so you can just get rid of all those trees.**

*We have removed the phylogenetic trees.*

**Reviewer #3: Fig 2.  there should be biological replicates for rigour**

**Fig 3 and throughout- statistical analysis are needed where conclusions are drawn about presence/absence of effect.**

*We now ensure that we have 3 biological replicates at the minimum for all the data presented in the paper. We have also updated all figures and text to include statistical analysis where needed and are now more precise in how we describe presence/absence of effect.*

--------------------

**Results**

**Reviewer #1: No, again, we describe more below. The authors should consider using figures as presentation of one pathway or process. Some cross pathways and are more difficult to understand. Additionally, some alleles are explained and some are not (in figures). Parallel data presentations and conclusions would make the manuscript more interpretable.**

*For consistency in data presentations, we have now updated the relevant main figures to have a) be the pathway, b) be the movement assay, c) the alignment and d) the structure. Accordingly, we have split Figure 2a into hypoxia (2b) vs ATP/nutrient-sensing (2f) pathways.*

*We apologise that we were not consistent in describing the alleles and have updated our figure legends to include additional allele information. For example, we now describe in the figure legends the nature of the different alleles used for aak-2 and rict-1:*

*“aak-2(ok524) is a loss-of-function mutant with a 409-bp deletion [59], while aak-2(rr48) is a semidominant-negative mutant with a substitution mutation [61]. Both rict-1(ft7) and rict-1(mg360) are loss-of-function mutants. rict-1(ft7) has a nonsense allele [66] while rict-1(mg360) has a missense allele [65].”*

*For Figure 5 we have created an additional table which explained the previous papers which had hypothesized the involvement of each of the individual genes in RQ-dependent metabolism. We hope these changes now make the pathways we are describing clearer.*

**Reviewer #2: 365  "We have thus identified two excellent targets for potential anthelmintics in the RQ synthesis pathway: COQ-2 and TDO-2." I see the data for TDO-2 in figure 3 but where is the data for COQ-2?**

*We have changed the wording to reflect that the COQ-2 target was identified by us in a previous paper and that we have furthered its potential as a target in this paper in former Figure 6. This sentence has now been re-written as:*

*“We have thus identified two excellent targets for potential anthelmintics in the RQ synthesis pathway: COQ-2 from our previous study [34] and TDO-2 as we describe here.”*

**Reviewer #3: line 480- 'data not shown'- the evidence should be provided**

**why in some cases is the KCN effect on motility shown and in others the recovery of motility following KCN exposure? Would it not be better to show both time-courses in all cases?**

*We apologize to the reviewer for not including the data in the original manuscript - that was an error on our part. We have now included the figure we alluded to in ‘data not shown’ in a supplemental figure (S11 Fig) and have shown that there is no significant difference between the single gene mutation or RNAi and the double combination. We have also included as supplemental figures (S2 Fig, S10 Fig) the corresponding time-courses for all the boxplot data presented in Figures 2 and 4.*

--------------------

**Conclusions**

**Reviewer #1: In some cases, yes. As we describe below, the authors can mention undersampled and preliminary conclusions. Additionally and importantly, the C. elegans results are massively oversold to all STH and even all parasitic nematodes. The results do not require that degree of salesmanship.**

*We apologise for appearing to oversell our conclusions. This was largely unintentional and is the result of our lack of precision in using STH throughout in a loose and imprecise manner. We apologise for this. We have also adjusted the wording in the paper to reflect the limitations of this study, for e.g.:*

*“We note that throughout this paper we use C. elegans as a model for STHs specifically as regards RQ-dependent metabolism though recognise that there are many biological differences between a free-living nematode and a parasitic helminth. Nonetheless it is the closest related model organism to STHs and the fundamental process of RQ synthesis and RQ dependent metabolism is likely to be largely conserved between these related species.”*

*“We do this using an assay that we previously described that allows us to assess the ability of C. elegans to use RQ-dependent metabolism [33]. Given the evolutionary relatedness between C. elegans and STHs, we think it likely that their closely related orthologues carry out similar roles in RQ-dependent metabolism in parasites”*

*And similar changes throughout and we hope that this now satisfies the reviewer*

**Reviewer #3: It is rather overly speculative in places, but nonetheless, I found it a very readable and intriguing account of the possibility that components of this pathway may present novel anthelmintic targets with good selective toxicity. The evidence for the involvement of the AMPK/mTOR pathway in sensing hypoxia is strong but could be substantiated by at least one rescue experiment.**

*We appreciate the reviewer has asked for a rescue experiment, but we note that the alleles that we used to test the involvement the AMPK/mTOR pathway are previously published and characterized and that we used two independent lines carrying to independent loss of function alleles for aak-2 and also for rict-1. And for each, the two mutants show a very similar response to KCN across a range of concentrations. While we have not done any rescue experiments, it seems unlikely to us that all 4 of these independent alleles are all giving the same artifactual result. We also note that rescue experiments often are fraught with artefacts themselves (overexpression, multicopy transgenes and the like) and thus the use of multiple independent alleles to confirm a genotype-phenotype connection is the standard in the C. elegans community. We hope the reviewer understands why we took this approach. Finally, to clarify — we do not believe that the AMPK/mTOR pathway is sensing hypoxia at all rather that it senses the metabolic consequences of anoxia i.e. the change in AMP to ATP ratios. This is the known role for this pathway.*

--------------------

**Editorial and Data Presentation**

**Reviewer #1: C. elegans as a helminth. It really depends on who you ask. Some helminth parasitologists will bristle at this contention. Again, the results do not require that level of overselling.**

*We have adjusted our wording throughout to reflect the status of C. elegans as a nematode. We did not mean to be contentious or to over claim here, this was simply poor wording on our part and we apologise.*

**Throughout some figures, the C. elegans convention is to italicize gene names with a hyphen between the three or four letter gene designation and the number. For example, dpy-10. The authors are not consistent about italics or the use of a hyphen in several figures.**

*We have been sure to be sure to adjust our figures and text to follow C. elegans gene naming conventions and thank the reviewer for spotting this.*

**Line 44: “resistance” should be clarified as “anthelmintic resistance”.**

*We have added the word anthelmintic to the sentence.*

**Line 52: Suggest changing “some of this” to “some of these changes involve”.**

*We have replaced “this” by “these”.*

**Line 86: Authors appear to be speaking more generally here; suggests making “host and STH” plural.**

*We have made “host and STH” plural.*

**Line 107-108: “previously described” Citation?**

*We have now added the citation.*

**Table 1: There is no legend indicating what the colors/+ indicate**

*We apologize that we did not make clear what the colours and + indicate. We are now more precise in what these denote and have indicated these in the figure legends. We have now added this legend:*

*“Candidate genes are grouped by category and the perturbation(s) is identified for each. Perturbations which resulted in a 50% reduction in movement at the median of all reps are marked with a + and coloured yellow, those which resulted in a reduction in movement of 25% at the median are marked with a ++ and coloured blue. Text is in underlined if the difference was 1.00e-02 < p <= 5.00e-02, in italics if it was 1.00e-03 <p <= 1.00e-02, in bold if it was 1.00e-04 <p <= 1.00e-03 and in bold-underlines if it was p <= 1.00e-05, all according to Welsh’s t-test. Active site changes were marked with a + and coloured yellow if S3Det identified residues which were in the vicinity of the active site and with a ++ and coloured blue if those residues were close to the active site and were a significant amino acid change.”*

**Line 281-283: Consider simplifying the sentence beginning with “Two of these…”**

*We have split the sentence in two for simplicity.*

**Line 290-291: Consider avoiding the absolute. “No high confidence target from our candidate genes”.**

*We specify our candidate ‘sensor’ genes now.*

**Line 463: Suggest inserting a colon after including to make the list more distinct.**

*We do not think a colon is necessary here and prefer it without if the reviewer is OK with this small stylistic difference.*

**Line 539: Identify but what about synthesize?**

*Of course we recognize that many compounds that might be identified as in silico potential inhibitors may be hard to synthesize at scale and at economically viable cost. The focus of this paper however is in finding initial leads rather than in the economics or synthesis of those leads which is an entirely distinct problem. We hope the reviewer appreciates that our study was always designed to focus on the identification of potential targets which is still many steps from a final viable anthelmintic drug in the real world and we acknowledge this difference.*

**Figure 2a: Y-axis scale could be shortened to allow more room to visualize some of the smaller boxes.**

*We understand this but still feel it is better to have a single y-axis across all data in this format since otherwise readers can sometimes draw incorrect conclusions by comparing data with different y-axes and hope the reviewer accepts this.*

**Figure 3d: Structure completely missing.**

*Thank you and we have addressed this.*

**Supplemental Table 1: For PHX769 and 243, the color coding of mutations is not actually highlighted on the sequence change. Additionally, extend the column width for strain, as it appears strain names are pushed to two lines.**

*We thank the reviewer for noticing this oversight. The colour coding we had in the original Word document was not transferred during the PDF build and we have now fixed this to ensure the sequence changes remain highlighted as indicated. We have also adjusted the column widths accordingly.*

**Reviewer #2: 'Helminth' is not a synonym for 'nematode'. A helminth is any wormlike parasite of interest to humans and may include, in addition to roundworms/nematodes, flatworms. C. elegans is not a parasite, and therefore not a helminth (line 83). Thus helminth is also not a phylogenetic classification. Comparing 'helminths' and annelids is not meaningful because annelids are a phylogenetic classification and 'helminths' are poly-phyletic. Since you seem to use helminth as a synonym for nematode, I would just use nematode, and explain to the reader that while the helminths you are interested in (the one for which C. elegans is a suitable model) are nematodes, not all nematodes are parasites, C. elegans is not a parasite, but it’s a good model for parasitic nematodes because it shares with parasites the switch to rhodoquinone under anoxic conditions.**

*We thank the reviewer for correcting this and acknowledge that we were careless in our use of ‘helminth’ for C. elegans. We have adjusted our wording to reflect this in line 83:*

*“we previously showed that these STH-specific inhibitors can efficiently kill C. elegans, a free-living Clade V nematode that is related to STHs, when it requires RQ-dependent metabolism to survive”*

*We have also added a Fig1a which shows the evolutionary relationship between the species which were studied in this paper as well as which clades each of the nematode species belong to. We now also define this clearly in the results section:*

*We next examined each amino acid sequence to identify whether there were specific residues that are identical across the helminths studied (Fig 1a) and that differ from those in a selection of their mammalian hosts (Fig 1a). Helminth species were selected so that 4/5 clades were represented and so that platyhelminthes were also included in the form of Fasciola hepatica (Fig 1a). We also included two molluscs and an annelid as their own group since they are also known to produce RQ [reviewed in 24]. Residues were considered of interest if they were conserved amongst the helminth (nematode +platyhelminth), C. elegans and mollusc+annelid group and were differentially conserved in mammalian hosts. We also checked whether any of those ‘helminth-specific’ residues reside at or near the active site.*

**2. By 'hosts' you seem to mean mammals of the human or livestock variety so say that. Not all hosts are mammals - nematodes parasitize virtually every metazoan phyla - so it doesn't make sense to compare 'hosts' to molluscs (which are also hosts to many nematode parasites). (line 384 'These are the three sets of animals that are..', also 511)**

*We have adjusted our wording to now more specifically refer to ‘mammalian hosts’ throughout and apologize that we were lax in defining ‘hosts’ as well as adjusting our wording on line 165 to ‘a selection of their mammalian hosts’.*

**Reviewer #3: The references need tidying up- for example spurious use of capitalisation- lack of italics where required**

*We have tidied up the references.*

**line 329 C. elegans,  in italics**

**Line 32 'identify' not 'identity'**

**throughout C. elegans (in italics) not C.elegans**

*We have corrected these and thank the reviewer for spotting these.*

--------------------

**Summary and General Comments**

**Reviewer #1: The authors identify potential druggable targets in rhodoquinone-dependent metabolic pathway in C. elegans and maybe other nematode species. Rhodoquninone has previously been established as necessary for helminth (as well as molluscs and annelids) metabolism in anaerobic environments, such as those inhabited by many soil transmitted helminths within hosts. Because rhodoquinone metabolism is not used in humans or livestock species, anthelmintics targeting this metabolic pathway are possibly nematode-specific. The authors have identified nine specific targets necessary for Rhodoquinone metabolism, and because all nine have human orthologues, worked to determine which targets or residues within these enzymes are helminth-specific. Although the work presented in this manuscript is interesting and offers important findings for the study of potential anthelmintic targets, this paper suffers from overall issues with logical flow and overinterpretation of the data. This manuscript took us (a molecular geneticist and a parasitologist) several reads and many hours to break down the results for this review. It should not take that much work to understand a manuscript. Below are major points and suggestions for revision, followed by minor suggestions and specific edits.**

**(1) The authors overreach with the applicability of their results. Authors initially suggest focusing on targets that are differentially expressed between free-living and parasitic life stages.**

*We are a little confused here — we did not suggest this nor are any of our targets differentially expressed between these stages. We are unsure how to address this since it is not part of this paper. The expression of the targets is not the issue — it is the requirement for them that changes and in some cases the specificity due to alternative splicing (COQ-2) or the directionality of the reaction (Complex II and ETFDH) but expression was not considered or mentioned anywhere in the paper.*

**They demonstrate that these potential targets are effective at inhibiting RQ-dependent metabolism in C. elegans, a Clade V nematode. Two out of three example STHs presented in the introduction (Ascaris and whipworms) do not have free-living life stages in the environment. Differences between clades and life cycle biology limit the applicability of these targets to all STHs, and further testing would be necessary to confirm these findings for other clades. An example of such differences can be found in resistance to benzimidazoles in ascarids. Resistance to BZ in Clade V parasitic nematodes has been well documented to be associated with mutations at codons 167,198, and 200 in beta-tubulin. Kruken et al. found that in A. lumbricoides, efficacy of mebendazole was significantly reduced, but found no resistance-associated mutations across the beta-tubulin genes. Differences between clades may play important roles in how treatments that affect these potential targets will affect different species.**

*We agree that we overstated the applicability of some of our conclusions and agree that our results may not be applicable across all parasitic helminths and have tried to reword this more carefully and tone down the conclusions throughout. We hope this satisfies this reviewer.*

**There is also a lack of consideration of how pharmacokinetics may impact these proposed targets. The authors cite beta-tubulin as an anthelmintic target that is highly similar between parasites and hosts, but treatment remains parasite-specific and is safe for hosts. Although it is true that slight variation is thought to be the deciding factor in BZs not targeting host tubulins, pharmacokinetics within the host are also thought to play an important role.**

*We disagree here. Pharmacokinetics has no relevance to a target but is critical for a drug. Our goal here was to identify which of a large possible set of enzymes *might* be good targets for anthelmintics and we identified the small subset that meet the criteria of having active sites that differ between parasite and mammalian host and that are also required for RQ dependent metabolism in our C. elegans model. Of course we are not claiming that every single compound predicted to inhibit the active sites of these targets in a species-selective manner will have perfect pharmacokinetics nor did we say this at any point of the paper. This was simply a first step towards identifying lead compounds and we do not think that a long discussion of pharmacokinetics is relevant in a study on target identification.*

**Although this work offers important new information on potential anthelmintic targets, data are overinterpreted to apply to all STHs, and assumes safety for hosts solely dependent on minor differences in proteins. The authors should do a substantial edit and reframing of their results. We believe that the C. elegans results (if improved as suggested below) can stand on their own and be important to present to the broader parasitology and NTD communities. The authors do not need to oversell their results to all STH and diverse nematode species.**

*We absolutely agree with the reviewer here and apologise for overstating the applicability of our conclusions. We were very lax with our use of the terms nematode and helminth and agree that we overstated our case. We have tried to tone this down and be more precise throughout. For example, we now summarise and frame our all results first and foremost in the context of C. elegans biology:*

*“By screening 25 candidate genes in our RQ-dependent metabolism assay, we aim to find a set of genes that are essential for C. elegans to carry out anaerobic RQ-dependent metabolism and suggest that their orthologues will act similarly in parasites.”*

**(2) Significant edits in writing are suggested to improve clarity and readability. The Results section is filled with previously published data, better suited for an introduction. These paragraphs make the flow harder to interpret and new results are lost in the mix. Results should focus more on what was found in this work. Although these previous findings are necessary to understand what led to the current work, the Results section should be focused on the unpublished data. The background information can be included in the Introduction, first figure (as it is now), and in summary figure panels for each pathway/process.**

*This is a hard balance to strike and we note that other reviewers actually asked us to add *more* previously published data. We have tried moving this all to the introduction but found that the flow was more confusing and thus have kept it more or less as previous but do appreciate the structural issues that they raise and have done our best to address the diverse opinions of the reviewers.*

**The order of data and figure presentation also makes this paper difficult to follow. Results within one section and/or figure focus in and out of the different pathways. It is suggested that authors go layer by layer discussing the results more generally and then focusing on the specific targets in each pathway. For example, Figure 2 focuses on hif-1 results in part of A, and all of B, C, and D. The authors can remove the extra pathway data from Figure 1A and focus that figure and data discussion solely on hif-1. Other figures can be focused on specific pathways.**

*We have now separated Figure 2 into two groups of figures, one for hypoxia and one for anoxia.*

**(3) Figures should be able to stand alone, and currently figures are difficult to interpret. It is suggested that authors first show the pathway with the steps and genes of interest being shown for each pathway. For example, first show a model with all components in panel A and then follow with the experimental results for subsequent panels For the audience of PLoS NTD, the authors need to provide a more understandable flow, data presentation in the figures, and writing. Otherwise, the manuscript will not have a significant impact on this community because of its lack of clarity.**

*We have adjusted the figures as suggested so as to always include a summary of the pathway and hope this helps. We do note that other reviewers said it was very readable so again we are trying to thread the needle between diverse reviewers opinions and hope that this goes a good way to resolving this.*

**(4) For results detailed in Figure 2a, there are not enough replicates to make strong conclusions. For most mutants tested, there are only 2-4 biological replicates. More data points are needed to make strong conclusions. Specifically, you can calculate a mean or median from two data points, but no statistician (or rigorous biologist) will ever believe the result. The authors should replicate the results or present the caveats of the under-replicated experiments.**

*We have removed the gene with two biological replicates and ensured that all data points have a minimum of three biological replicates. We have included statistics on all the movement plots and made it clear that each biological replicate is the average of multiple technical replicates, each of which reflect the movement of ~150 worms. We thank the reviewer for this point and completely agree that statistics were needed to strengthen the conclusions we make.*

**Minor Points:**

**(1) The authors should be more detailed in lines 41-47. The authors include levamisole with these other two drugs in the discussion but neglect to include it here. Resistance is widespread in livestock, but not in the nematode groups included here. To our knowledge, many of the studies in humans reporting lack of efficacy have potential confounding factors as briefly discussed in the Moser et al. cited here. It would be more accurate to say that in some regions, there are reports on lack of efficacy although resistance is not definitive.**

*We have reworded this section to make it more clear and included levamisole as an essential drug in line 43.*

**(2) Although not consistently used, there are a number of statements the authors use repeatedly but somewhat inaccurately. Authors refer to STH-specific active sites, but all work is done in the model nematode C. elegans, which is not an STH. Be more general with the language. Also what about non-STH parasitic helminths such as Brugia malayi? This would be worth at least briefly mentioning. As stated above, we believe the C. elegans results are interesting and important to present without overselling the relevance as broad STH treatments.**

*We thank the reviewer for this and absolutely agree that we were very lax in our terminology, using C. elegans a nematode and STH almost interchangeably and that was a bad error on our part. We have changed our wording throughout and hope this resolves many of these issues.*

**(3) In the discussion, the authors suggest that treatments targeting their identified enzymes should act to prevent RQ-dependent metabolism, leading to parasite death. Before this section, the authors were more dogmatic. For example, in line 66, “would thus kill”. The more speculative “should” works better in this context, as nothing can be known for sure until therapeutics that target these sites are actually used.**

*We have replaced “would” by “should” in line 66.*

**(4) All assays with C. elegans were done using the L1 life stage, after exposure to cyanide. How did this affect development in your strains? We would consider addressing the possibility of differences between the C. elegans life stages used here and those of the STHs infecting hosts in the Discussion. Parasitic nematodes develop within their eggs to later life stages. Have you considered possible differences in effects across these different life stages?**

*We have now included supplementary data (S1 Fig) showing the differences in recovery in response to KCN in all larval stages between N2 and kynu-1(e1003). All stages show paralysis after prolonged KCN treatment and all the stages in N2 animals are able to recover while the kynu-1(e1003) animals do not, suggesting that the worms can also switch to using RQ-dependent metabolism in those stages. Therefore, we think that our findings in L1 worms should be applicable to other life stages though we appreciate that there may be differences between the free-living C. elegans and parasitic nematodes and have now noted this in the text:*

*“We note that throughout this paper we use C. elegans as a model for STHs specifically as regards to RQ-dependent metabolism though recognise that there are many biological differences between a free-living nematode and a parasitic helminth.”*

**(5) As Figure 5 is showing negative results, it is suggested that it is moved to the supplemental figures, and indicated as negative results within the text.**

*While Figure 5 shows negative results, we have not moved it to the supplemental figures as we describe the results quite extensively in the text and feel it is better suited as a main figure.*

**(6) In Figure 6, Panel A is a gene model for coq-2 but not other potential targets have gene models. This specificity again distracts from the flow. We suggest adding the full gene model (not just four exons) to the supplement. As suggested above, Figure 6A could be a model of the pathway. In previous figures, genes were named simply with the mutant allele, and it is suggested this is made consistent with this figure.**

*We have also now renamed the strains with their allele names to be consistent in how we present the data and thank the reviewer for this comment. The reason that we show a gene model for coq-2 is that the key to this gene is a change in alternative splicing and the gene editing that we did was specifically to interfere with that alternative use of two exons. Without a gene model this would be very hard to follow. However no such alternative splicing is relevant in any of the other targets and thus it seems unnecessary to show it for them. We hope the reviewer appreciates this.*

**Reviewer #2: The manuscript would greatly benefit from strict editing. The rationale for focussing on the rhodoquinone pathway is belabored at several points in the manuscript as is the role of the reversal of electrode transport (e.g. paragraph line 445). The discussion is nearly fully redundant with text in the results section. I would encourage the authors to instead use the discussion to address wider implications such as, why are Complex II drugs so easy to find (almost every  drug screen in C. elegans finds one) even though the drugs are not screened under anoxic conditions? Don't your results mean that we should be doing drug screens in C. elegans under anoxic conditions to find anoxia-specific drugs? Just some examples off the top of my head.**

*We have rewritten sections of the paper in an attempt to address these issues. We also respectfully disagree with the reviewer regarding Complex II. We have undertaken large scale drug screens and while we do indeed find one novel Complex II inhibitor, we find many more compounds that inhibit other complexes of the electron transport chain as well as many that act as ionophores and collapse the proton motive force as well as a host of compounds affecting the nervous system and other metabolic inhibitors. We simply disagree with the comment and don't understand this — it is far from our experience and thus feel that discussing this would be inaccurate and speculative. We also are finding hard to fit this comment with the view of Reviewer 3 that it was "very readable" so have left the discussion more or less the same. Regarding the anoxia conditions, as we make clear in the paper we believe that this is *exactly* what we are simulating with our KCN treatment and we do it using KCN since working in an anoxia chamber is cumbersome and costly for large scale drug screens.*

**Minor stuff**

**230 and 4.5 hours. Worms incubated in M9 buffer alone was... (were)**

*We have replaced ‘was’ by ‘were’.*

**Fig 2. Are you sure high concentrations of KCN don't quench GFP - just a thought.**

*We have now included in the supplemental figures (S3 Fig) RNA-seq data showing that nhr-57 expression is not increased after 3h or even after 15h of 200 µM KCN treatment, which corroborates our GFP reporter data and suggests the lack of fluorescence in high KCN is unlikely due to quenching by KCN.*

**Reviewer #3: This is a very interesting paper of considerable breadth investigating hypoxic metabolism in C. elegans. It is rather overly speculative in places, but nonetheless, I found it a very readable and intriguing account of the possibility that components of this pathway may present novel anthelmintic targets with good selective toxicity.**

**I disagree with the statement on line 631-line 636 – cite ivermectin as example of anthelmintic that targets invertebrate unique ion channel GluCl. also monepantel targets a unique nicotinic channel.**

We agree with reviewer on this point and acknowledge that we were not careful with our wording of known anthelmintic targets. We have now edited this section to include ivermectin and monepantel as such examples and thank the reviewer for noting this. This section is now re-written as:

*Unexpectedly, while helminth genomes contain many helminth-specific genes and gene families, few of these has emerged as a target of major anthelmintics to date and anthelmintics typically target proteins that are shared between parasites and their mammalian hosts. We note ivermectin and monepantel as exceptions to this – ivermectin targets glutamate-gated chloride ion channels that are not present in vertebrates while monepantel target the nematode-specific DEG-3 subfamily of acetylcholine receptors. However, in most cases, the selectivity of anthelmintics for helminths is due to subtle differences between the helminth and host protein sequences.*

**It is rather long and somewhat repetitious in places and could be edited for brevity.**
